# Supplementary material for: MetaRibo-Seq measures translation in microbiomes
Source: Nat Commun. 2020 Jun 29;11:3268. doi: 10.1038/s41467-020-17081-z (PMC7324362; doi:10.1038/s41467-020-17081-z)
Supplement: Supplementary file 10 — Supplementary Data 7 [file 41467_2020_17081_MOESM10_ESM.zip › File2/Confidence_High_Taxonomy/83481_out.krona.html]

Javascript must be enabled to view this page.

members
magnitude
magnitudeUnassigned
count
unassigned
taxon
rank

83481\_out

167

2
superkingdom
167

phylum
167
976

200643
167
class

171549
order
167

family
2
1853231

574697
1
genus

1472416
species

SRS014613\_contig\_number\_13322
1

283168
1
genus

2292048

SRS075341\_contig\_number\_23731
1
species

815
family
165


SRS014923\_contig\_number\_contig-100\_958.242694SRS046712\_contig\_number\_1859SRS065504\_contig\_number\_11082SRS077127\_contig\_number\_10499SRS097920\_contig\_number\_16281SRS142980\_contig\_number\_7162SRS143876\_contig\_number\_15539SRS144537\_contig\_number\_45416
8
816
genus
165

457389
2

SRS049402\_contig\_number\_1590SRS103987\_contig\_number\_12759
species

338188

SRS103987\_contig\_number\_19167
1
species

469590
species

SRS055982\_contig\_number\_18822SRS063190\_contig\_number\_14477
2

1739298
species
1

SRS013638\_contig\_number\_10023

585544
species
1

SRS063370\_contig\_number\_7809

2

SRS1041136\_contig\_number\_17968SRS148159\_contig\_number\_5432
species
817

species

SRS023526\_contig\_number\_17238
1
1262750

2292956

SRS015578\_contig\_number\_25821SRS019601\_contig\_number\_13737SRS022137\_contig\_number\_18938SRS074670\_contig\_number\_2543SRS1041142\_contig\_number\_2878SRS143466\_contig\_number\_29526SRS147977\_contig\_number\_1683
7
species

species

SRS014412\_contig\_number\_879SRS149075\_contig\_number\_18754SRS893259\_contig\_number\_4735
3
46506

818
species

SRS024132\_contig\_number\_31068SRS049773\_contig\_number\_908SRS143148\_contig\_number\_26133SRS143372\_contig\_number\_7653
4

species
1

SRS045244\_contig\_number\_8415
2292957

371601
32

SRS016989\_contig\_number\_contig-100\_1513.95325SRS017247\_contig\_number\_2732SRS017521\_contig\_number\_30720SRS018049\_contig\_number\_1003SRS019286\_contig\_number\_1850SRS020869\_contig\_number\_13610SRS021153\_contig\_number\_2202SRS043411\_contig\_number\_11932SRS043841\_contig\_number\_11206SRS045713\_contig\_number\_8995SRS049446\_contig\_number\_10527SRS056537\_contig\_number\_64917SRS075078\_contig\_number\_1139SRS075716\_contig\_number\_3942SRS098073\_contig\_number\_3249SRS101268\_contig\_number\_1643SRS1041036\_contig\_number\_1954SRS1041130\_contig\_number\_4393SRS1041144\_contig\_number\_6313SRS1041146\_contig\_number\_776SRS104311\_contig\_number\_18731SRS104636\_contig\_number\_25404SRS1055038\_contig\_number\_2249SRS1055067\_contig\_number\_15828SRS1055099\_contig\_number\_10918SRS143181\_contig\_number\_12261SRS148091\_contig\_number\_12776SRS148196\_contig\_number\_11889SRS148721\_contig\_number\_53628SRS149325\_contig\_number\_12563SRS149879\_contig\_number\_16032SRS893366\_contig\_number\_18934
species

species

SRS045195\_contig\_number\_7405SRS046068\_contig\_number\_4813
2
821

3

SRS1041164\_contig\_number\_1638SRS143523\_contig\_number\_3713SRS893295\_contig\_number\_9795
species
47678

species
6

SRS019787\_contig\_number\_1592SRS024663\_contig\_number\_14574SRS050752\_contig\_number\_25769SRS051610\_contig\_number\_2034SRS077730\_contig\_number\_3775SRS1055056\_contig\_number\_13971
457387

species
70

SRS011302\_contig\_number\_25739SRS012849\_contig\_number\_17412SRS012902\_contig\_number\_190SRS013215\_contig\_number\_315SRS013382\_contig\_number\_contig-100\_1024.28381SRS013476\_contig\_number\_contig-100\_561.225651SRS014287\_contig\_number\_1612SRS014979\_contig\_number\_3803SRS015065\_contig\_number\_37020SRS015369\_contig\_number\_6418SRS015578\_contig\_number\_36343SRS015854\_contig\_number\_1241SRS016840\_contig\_number\_862SRS018817\_contig\_number\_2624SRS018936\_contig\_number\_11420SRS019030\_contig\_number\_14436SRS019381\_contig\_number\_3392SRS019397\_contig\_number\_9420SRS019968\_contig\_number\_16796SRS022713\_contig\_number\_2486SRS023346\_contig\_number\_824SRS023829\_contig\_number\_98SRS024388\_contig\_number\_6559SRS024549\_contig\_number\_4407SRS043701\_contig\_number\_16751SRS045004\_contig\_number\_30441SRS046369\_contig\_number\_16231SRS046717\_contig\_number\_4250SRS049896\_contig\_number\_27160SRS049959\_contig\_number\_52465SRS053398\_contig\_number\_25279SRS053573\_contig\_number\_676SRS056259\_contig\_number\_1944SRS057478\_contig\_number\_5426SRS057717\_contig\_number\_134SRS058268\_contig\_number\_122SRS063813\_contig\_number\_6095SRS064232\_contig\_number\_1689SRS064276\_contig\_number\_5530SRS075398\_contig\_number\_728SRS077086\_contig\_number\_12398SRS077231\_contig\_number\_20184SRS077502\_contig\_number\_14435SRS098061\_contig\_number\_13233SRS098881\_contig\_number\_6013SRS1041092\_contig\_number\_4174SRS1041095\_contig\_number\_contig-100\_337.34434SRS1041137\_contig\_number\_32473SRS1041139\_contig\_number\_5996SRS1041147\_contig\_number\_4869SRS104400\_contig\_number\_31247SRS105153\_contig\_number\_12990SRS1055095\_contig\_number\_1052SRS140645\_contig\_number\_90SRS143598\_contig\_number\_2115SRS143895\_contig\_number\_45872SRS147346\_contig\_number\_52751SRS147377\_contig\_number\_11983SRS147425\_contig\_number\_11203SRS147653\_contig\_number\_6168SRS148511\_contig\_number\_18189SRS148784\_contig\_number\_25106SRS148970\_contig\_number\_3666SRS893256\_contig\_number\_2077SRS893285\_contig\_number\_2175SRS893292\_contig\_number\_2394SRS893327\_contig\_number\_10862SRS893342\_contig\_number\_8650SRS893369\_contig\_number\_3160SRS893378\_contig\_number\_22510
28116

2

SRS014459\_contig\_number\_20456SRS015190\_contig\_number\_10593
species
246787

1739517

SRS014736\_contig\_number\_2451SRS016056\_contig\_number\_1678SRS022524\_contig\_number\_8183SRS023583\_contig\_number\_8756SRS046810\_contig\_number\_contig-100\_51.52SRS054956\_contig\_number\_14059SRS056519\_contig\_number\_1502SRS064557\_contig\_number\_11064SRS076756\_contig\_number\_15719SRS101376\_contig\_number\_15721SRS1041138\_contig\_number\_10870SRS1041141\_contig\_number\_9521SRS140513\_contig\_number\_10893SRS142923\_contig\_number\_16135SRS146813\_contig\_number\_28792SRS147022\_contig\_number\_2152SRS893373\_contig\_number\_25587
17
species
